# The CpxA/CpxR Two-Component System Affects Biofilm Formation and Virulence in *Actinobacillus pleuropneumoniae*

**DOI:** 10.3389/fcimb.2018.00072

**Published:** 2018-03-20

**Authors:** Huan Li, Feng Liu, Wei Peng, Kang Yan, Haixu Zhao, Ting Liu, Hui Cheng, Peixi Chang, Fangyan Yuan, Huanchun Chen, Weicheng Bei

**Affiliations:** ^1^State Key Laboratory of Agricultural Microbiology, College of Veterinary Medicine, Huazhong Agricultural University, Wuhan, China; ^2^The Cooperative Innovation Center for Sustainable Pig Production, Huazhong Agricultural University, Wuhan, China; ^3^Key Laboratory of Development of Veterinary Diagnostic Products of Ministry of Agriculture, Huazhong Agricultural University, Wuhan, China; ^4^Hubei Key Laboratory of Animal Embryo and Molecular Breeding, Institute of Animal Husbandry and Veterinary Sciences, Hubei Academy of Agricultural Sciences, Wuhan, China

**Keywords:** *Actinobacillus pleuropneumoniae*, CpxA/CpxR, biofilm, *rpoE*, virulence

## Abstract

Gram-negative bacteria have evolved numerous two-component systems (TCSs) to cope with external environmental changes. The CpxA/CpxR TCS consisting of the kinase CpxA and the regulator CpxR, is known to be involved in the biofilm formation and virulence of *Escherichia coli*. However, the role of CpxA/CpxR remained unclear in *Actinobacillus pleuropneumoniae*, a bacterial pathogen that can cause porcine contagious pleuropneumonia (PCP). In this report, we show that CpxA/CpxR contributes to the biofilm formation ability of *A. pleuropneumoniae*. Furthermore, we demonstrate that CpxA/CpxR plays an important role in the expression of several biofilm-related genes in *A. pleuropneumoniae*, such as *rpoE* and *pgaC*. Furthermore, The results of electrophoretic mobility shift assays (EMSAs) and DNase I footprinting analysis demonstrate that CpxR-P can regulate the expression of the *pgaABCD* operon through *rpoE*. In an experimental infection of mice, the animals infected with a *cpxA/cpxR* mutant exhibited delayed mortality and lower bacterial loads in the lung than those infected with the wildtype bacteria. In conclusion, these results indicate that the CpxA/CpxR TCS plays a contributing role in the biofilm formation and virulence of *A. pleuropneumoniae*.

## Introduction

*Actinobacillus pleuropneumoniae* is a species of Gram-negative bacteria that causes porcine contagious pleuropneumonia (PCP), which is characterized by fibrinous pleurisy and fibrinous pneumonia. Each year this pathogen causes substantial economic damages in the pork industry globally (Zimmerman et al., 2012). Previous studies have discovered many virulence factors of *A. pleuropneumoniae* (Fuller et al., 2000; Sheehan et al., 2003). These virulence factors, such as Apx toxins, capsule, adhesins, transferrin-binding proteins, and outer membrane proteins, play crucial roles in the pathogenicity of *A. pleuropneumoniae* (Chiers et al., 2010).

To increase their adaptability, bacteria generally utilize two-component signal transduction systems (TCSs) to perceive and respond to environmental influences. A representative TCS consists of a histidine kinase (HK) in the inner membrane and a response regulator (RR) in the cytoplasm (Buelow and Raivio, 2010). After perceiving an environmental signal, the HK subsequently autophosphorylates and transfers the same phosphate group to the RR, after which the RR can influence the transcription level of downstream genes by binding to their promoter regions (Vogt and Raivio, 2012). Whole genome sequencing of *A. pleuropneumoniae* serovar 3 strain JL03 revealed five putative TCSs: ArcA/ArcB, QseB/QseC, CpxR/CpxA, NarP/NarQ, and PhoB/PhoR (Xu et al., 2008).

When it was initially characterized, the TCS CpxA/CpxR was viewed as a newly discovered envelope stress response system. Experimental evidence in *Escherichia coli* then provided a model: when misfolded envelope proteins accumulate, CpxA autophosphorylates and then transfers the phosphate group to CpxR, which causes the upregulation of a series of chaperonins and proteases that can degrade or refold the misfolded proteins, thus alleviating the envelope stress (Vogt and Raivio, 2012). In recent years, the CpxA/CpxR system has been demonstrated to be involved in the virulence of uropathogenic *Escherichia coli* (Debnath et al., 2013), *Salmonella enterica* serotype Typhimurium (Humphreys et al., 2004), and *Vibro cholerae* (Acosta et al., 2015). The CpxA/CpxR system has also been shown to be involved in the biofilm formation of *E. coli* (Dorel et al., 1999; Ma and Wood, 2009; Dudin et al., 2014). In addition, the CpxA/CpxR system has been implicated in antibiotic resistance (Mahoney and Silhavy, 2013).

Biofilms are aggregated bacterial groups that are embedded in a matrix of extracellular polymeric substances (Donlan and Costerton, 2002). Biofilm formation of *A. pleuropneumoniae in vitro* (such as on polystyrene microtiter plates) depends on the lipopolysaccharides (LPS), the capsular polysaccharides (CPS) and the yields of β-1,6-N-acetyl-D-glucosamine (PGA) polymer (Kaplan et al., 2004; Hathroubi et al., 2015b). In *A. pleuropneumoniae*, the production of PGA depends on the expression of the *pgaABCD* operon, which is repressed by the protein H-NS and upregulated by the alternative sigma factor σ^E^ (Bosse et al., 2010). Kaplan et al. found that only two of the fifteen *A. pleuropneumoniae* reference strains, including 5b and 11, were able to form a pronounced biofilm on polystyrene microtiter plates (Kaplan and Mulks, 2005). However, Hathroubi and colleagues found that when cultured in the presence of sub-minimum inhibitory concentration of penicillin G, many *A. pleuropneumoniae* strains can form pronounced biofilms and this is likely the result of a cell envelope stress sensed by the CpxRA system resulting in an increased production of PGA and other matrix components (Hathroubi et al., 2015a). Several studies have also proposed that CpxA/CpxR may be involved in the mechanism of biofilm formation in *A. pleuropneumoniae* (Tremblay et al., 2013; Hathroubi et al., 2015b). However, these studies did not use a deletion mutant to confirm the phenotype directly.

Although previous work has demonstrated that CpxA/CpxR plays an important role in biofilm formation and virulence in *E. coli* and other bacteria, the role of CpxA/CpxR in *A. pleuropneumoniae* remained unclear. In this study, we found new growth conditions that induce biofilm formation in *A. pleuropneumoniae* (i.e., 42°C, TSB, static). Based on this new system, we investigated the role of CpxA/CpxR in biofilm formation, focusing on the *rpoE* gene and its relationship to the expression of the *pga* operon. We also assessed whether or not CpxA/CpxR contributes to the virulence of *A. pleuropneumoniae* serotype 1 strain S4074.

## Materials and methods

### Bacterial strains and culture conditions

The bacterial strains, primers, and plasmids used in this study are listed in Table 1. *A. pleuropneumoniae* strains were cultured at 37°C or 42°C in tryptic soy broth (TSB; Difco Laboratories, Detroit, MI, USA) or on tryptic soy agar (TSA; Difco Laboratories) supplemented with nicotinamide adenine dinucleotide (10 μg mL^−1^) and 10% (v/v) newborn calf serum. Additionally, *A. pleuropneumoniae* strains were also cultured in brain heart infusion broth (BHIB; Oxoid Ltd., Hampshire, United Kingdom) or on brain heart infusion agar (BHIA; Oxoid Ltd.) supplemented with 15 μg mL^−1^ NAD (Solarbio, Beijing, China) at 37°C. For the selection of *A. pleuropneumoniae* transformants, chloramphenicol (5 μg mL^−1^) was added. When culturing different *E. coli* strains, appropriate antibiotics were added in Luria-Bertani (LB) broth or agar (Haibo, Qingdao, China). For cultivation of *E. coli* β2155, diaminopimelic acid (dapA; 50 μg mL^−1^) (Sigma-Aldrich, St. Louis, USA) was added to LB medium (Yuan et al., 2014).

**Table 1:** Bacterial strains and plasmids used in this study.

**Strains/plasmids**	**Characteristics**	**Source/references**
***A. pleuropneumoniae***		
*S4074*	*A. pleuropneumoniae* reference strain of serovar 1; WT strain	Dr P. Blackall
Δ*cpxAR*	*A. pleuropneumoniae* 4074 *cpxAR*-deletion mutant	This study
*C*Δ*cpxAR*	Complemented strain of Δ*cpxAR*; Cm^r^	This study
***E. coli***		
*DH5a*	Cloning host for recombinant vector	Takara
*BL21(DE3)*	The expression host for pET-28a and their derivative	Takara
*β2155*	Transconjugation donor for constructing mutant strain	From Prof. Gerald-F. Gerlach
**PLASMID**		
pMD18-T	T-vector; Amp^r^	Takara
pMD18-T-*cpxAR*	pMD18-T carrying *cpxAR* genes	This study
pMD19-T	T-vector; Amp^r^	Takara
pMD19-T-*rpoE*	pMD19-T carrying the promotor region of *rpoE*	This study
pEMOC2	Transconjugation vector: ColE1 ori mob RP4 sacB, Amp^r^Cm^r^	From Prof. Gerald-F. Gerlach
pEΔ*cpxAR*	Up- and down-stream arms of *cpxAR* were ligated sequentially into pEMOC2, and used as the transconjugation vector for *cpxAR* gene deletion	This study
pJFF224-XN	*E. coli*-APP shuttle vector: RSF1010 replicon; mob oriV, Cm^r^	Frey, 1992
pCΔ*cpxAR*	pJFF224-XN carrying the intact *cpxAR*	This study
pET-28a	Expression vector; Kan^r^	Novagen
pET-*cpxR*	pET-28a carrying *cpxR* gene	This study
pET-*rpoE*	pET-28a carrying *rpoE* gene	This study

*Cm^r^, Chloramphenicol resistance; Amp^r^, Ampicillin resistance; Kan^r^, Kanamycin resistance*.

### Construction of the *A. pleuropneumoniae* mutant Δ*cpxAR* and complement CΔ*cpxAR*

To construct a *cpxA/cpxR* (*cpxAR*) gene deletion mutant, the upstream and downstream fragments of these two genes were amplified from the genomic DNA of *A. pleuropneumoniae* strain S4074 (wildtype, WT) using primers *cpxAR*-up F/R or *cpxAR*-down F/R. These PCR products were cloned sequentially into the suicide vector pEMOC2 to generate plasmid pEΔ*cpxAR*. The mutant Δ*cpxAR* was then constructed using the plasmid pEΔ*cpxAR* as previously described (Liu et al., 2015).

For complementation studies, the intact *cpxAR* genes were cloned with their promoters from S4074 using primers *cpxAR*-F and *cpxAR*-R. The entire fragment was then ligated into the T vector pMD18-T, generating plasmid pMD18-T-*cpxAR*. For constructing complementation plasmid pCΔ*cpxAR*, the 2.3-kb fragment from pMD18-T-*cpxAR* containing the *cpxAR* genes was ligated into *E. coli–A. pleuropneumoniae* shuttle vector pJFF224-XN via *Pst*I–*Not*I restriction sites (Yuan et al., 2014). Then, pCΔ*cpxAR* was electroporated (2.5 KV, 25 μFD, 800 Ω) into the mutant strain Δ*cpxAR*. The correct CΔ*cpxAR* was selected via its chloramphenicol resistance. When the CΔ*cpxAR* strain was cultured, 2 μg mL^−1^ chloramphenicol was always added in the culture medium.

The resultant mutant strain Δ*cpxAR* and complement strain CΔ*cpxAR* were verified by both PCR amplification with primers *cpxAR*-exterior F/R and *cpxAR*-interior F/R and RT-PCR amplification with primers *cpxA*-F/R and *cpxR*-F/R. The sequence accuracy of each strain was verified by DNA sequencing (data not shown).

### Biofilm assay

The microtiter plate biofilm assay is especially advantageous for examining early processes in biofilm formation (Merritt et al., 2005). Overnight inoculums were balanced to the same optical density (OD) with fresh TSB, and 100 μL of the 1:100-diluted balanced inoculums were transferred to each well of a 96-well microtiter plate (Corning, USA). Following an incubation of 36, 48, or 60 h at 37°C or 42°C, each well was washed three times with 200 μL of sterile PBS to remove slackly adherent cells. To fix the remaining attached bacteria, 100 μL of methanol was added to each well. After air-drying, the wells were then stained with 100 μL per well of crystal violet (0.1%) for 10 min at room temperature. After removing the crystal violet solution, the wells were washed under running tap water and dried in a 37°C incubator for 30 min. At this point, the plates were photographed. Last, 100 μL per well of glacial acetic acid (33%, v/v) was added, and the OD_590nm_ of each well was monitored by a Multi-Detection Microplate Reader. Furthermore, we also used this method to detect the biofilm formation of *A. pleuropneumoniae* strains grown in BHIB, since this is the common method used inducing biofilm formation in *A. pleuropneumoniae* (Labrie et al., 2010). Both assays were performed in triplicate.

### RNA extraction, RT-PCR, and real-time RT-PCR

The WT (S4074), *cpxAR* mutant, and complement strains were grown in TSB in six-well plates for 4 h at 37°C or 42°C. Ice-cold methanol was used to prevent changes in transcript levels after the planktonic cells of each strain were collected (Subashchandrabose et al., 2013). A total of 1 mL of bacterial cells was blended with 1 mL of 100% ice-cold methanol and centrifuged at 10,000 × g for 3 min at 4°C. For real-time RT-PCR (qRT-PCR), total RNA from the WT, mutant, and complement strains was extracted using the Bacteria Total RNA Isolation Kit (Sangon Biotech, China) and was then reverse-transcribed using the HiScript II Q RT SuperMix for qRT-PCR (+gDNA wiper) (Vazyme, China) following the manufacturer's instructions. The real-time PCR experimental method was based on SYBR-Green dye, and all reactions were performed in triplicate. The 10-μL real-time PCR reaction mixtures contained 5 μL of 2× SYBR Green Master Mix (Vazyme, China), 0.2 μL of Rox Reference Dye 2, 0.2 μL each of the forward and reverse primers (10 μM), 1 μL of template cDNA, and 3.4 μL of ddH_2_O. The real-time PCR amplification conditions were as follows: 5 min at 50°C, then 5 min at 95°C, followed by 40 cycles of 10 s at 95°C and 35 s at 60°C. The ViiA™ 7 real-time PCR system was used for qRT-PCR analysis by the 2^−Δ*Ct*^ method (Pfaffl, 2001). For normalizing the relative expression of target genes, the 16S rRNA gene was used as an endogenous control (Hathroubi et al., 2015b). The cDNA of WT was also used for RT-PCR by primers *cpxAR*-RT-F/R.

### Expression of his-CpxR protein in *E. coli*

The *cpxR* gene was amplified via PCR from the genomic DNA of *A. pleuropneumoniae* S4074 using primers P*cpxR*-F and P*cpxR*-R (Table 2) and then cloned into the *Bam*HI and *Hin*dIII sites of prokaryotic expression plasmid pET-28a, generating the recombinant plasmid pET-*cpxR*; the accuracy of the inserted sequence was confirmed by DNA sequencing. The plasmid pET-*cpxR* was transferred into BL21 (DE3) strain and grown at 37°C with energetic shaking in 1 L of LB broth containing 50 μg mL^−1^ kanamycin to an OD_600_ of 0.6–0.8. The *E. coli* BL21 (DE3) cells containing the plasmid pET-*cpxR* were then induced by isopropyl-β -D-thiogalactoside (0.25 mM) at 25°C for 5 h. A Low Temperature Ultra-high Pressure Continuous Flow Homogenizer (JNBIO, China) was used to disrupt cells, and the resulting cellular debris and membranes were removed by centrifugation at 12,000 × g for 20 min at 4°C. The recombinant protein was purified by Ni-NTA resin affinity chromatography, as described in the OIAexpress manual (Qiagen, Germany). The purified protein rCpxR was dissolved by elution buffer and was stored at −80°C until use. The expression of His-RpoE was also performed as above described.

**Table 2:** Primers used in this study.

**Primers**	**Sequence (5′-3′)^a^**	**Source or references**
**FOR MUTANT CONSTRUCTION**		
cpxAR-up F/R	CGTCGACCCGTTCATAATCGTCATAGT	This study
	CCGTCTAGAGACCGCTTGTTTCTACTC	
cpxAR-downF/R	GGTCTAGAGTTCGTGCAGAGAGCA	This study
	TGCGGCCGCTTAATCGTTTCTTTGT	
cpxAR-exterior F/R	CGAACTTACGCTGACG	This study
	ATGGCGCAATACCCT	
cpxAR-interior F/R	CAGTGTAATAGCAAGTAAGATAGCG	This study
	GTCTCCGGAAGAAAATAGCAA	
**FOR COMPLEMENT CONSTRUCTION**		
cpxAR-F/R	AAAACTGCAGCAAACCTTGATAAAGTTGTAAATT	This study
	AAGGAAAAAAGCGGCCGCTTATTCAATCCATAAAGGTAACTT	
**FOR RT-PCR**		
cpxA-F/R	GATTTTGTTCGGCATCGAAT	This study
	CGGAATTAACTCGGATCGAA	
cpxR-F/R	CCGGAGACTGGTTGGAATAA	This study
	ATGAAATTGATCGCGTCCTC	
**FOR PROTEIN EXPRESSION**		
PcpxR-F/R	CGGGATCCATGCCTAGAATTTTACTCGTTG	This study
	GGGAAGCTTAAGGACTTATTTTTCAGTAACGAG	
PrpoE-F/R	CGCCCATATGATGAGTGAGCTGGTAGCCGATCAAG	This study
	CGCCCTCGAGAATCTGTTGCATTAGCGGATTG	
**FOR EMSA ASSAY**		
rpoE-F/R	TAAAAAGATAAGATAAGCGGTC	This study
	AGTGTGTAACAAAAATGAAAAGT	
pga-F/R	TAATTAAGACGTCCGACTTGCTTTA	This study
	GCATTCTTAATCGCATAAAGACTAC	
**FOR RT-PCR AND REAL-TIME PCR**		
16SrRNA-F/R	CCATGCCGCGTGAATGA	Subashchandrabose et al., 2013
	TTCCTCGCTACCGAAAGAACTT	
pgaC-F/R	GATTCCGCATTTGCTCAATCT	This study
	CAATACCGCATCACCGTCTATG	
rpoE-F/R	TTTGATGTTGGGGGTCAACT	This study
	TGCATCAATCGCTTCTCTTG	

a *Restriction sites are underlined*.

### Electrophoretic mobility shift assays

The promoter regions of *rpoE* and *pgaABCD* genes were cloned into the T-vector pMD19-T to generate pMD19-T-*rpoE* and pMD19-T-*pgaABCD*. The promoter regions of *rpoE* and *pgaABCD* were then amplified via PCR from the plasmid using primers M13F-47 (FAM) and M13R-48, generating fluorescent FAM-labeled probes.

The recombinant protein CpxR was phosphorylated by acetyl phosphate (Sigma, USA) (Pogliano et al., 1997). Electrophoretic mobility shift assays (EMSAs) were carried out in 20 μL of reaction buffer (50 mM Tris-HCl (pH 8.0), 2.5 mM MgCl_2_, 100 mM KCl, 2 μg of salmon sperm DNA, 0.2 mM DTT, and 10% glycerol) that contained 40 ng of probe and varied quantities of CpxR or RpoE proteins. After incubation for 30 min at 30°C, the reaction liquid was loaded onto 4% non-denaturing PAGE gels in 0.5× TBE [5.4 g L^−1^ of Tris base, 2.75 g L^−1^ of boric acid, 2 mL L^−1^ of 0.5 M EDTA (pH 8.0)]. The resulting gel was photographed using an ImageQuant LAS 4000 mini system (GE Healthcare, USA).

### DNase I footprinting assay

The DNase I footprinting assay was carried out as previously described (Wang et al., 2012). For each assay, 40 μL of the total reaction mixture contained 400 ng of the probe and different amounts of CpxR. After incubating for 30 min at room temperature, a 10-μL solution [0.015 units of DNase I (Promega, USA) and 100 nmol of freshly prepared CaCl_2_] was added to the mixture and incubated for 1 min at room temperature. To stop the reaction, 140 μL of DNase I stop solution, which contained 200 mM unbuffered sodium acetate, 30 mM EDTA, and 0.15% SDS, was added to the mixture. The processing method of samples and the DNA ladder, electrophoresis, and data analysis were all performed as previously described (Wang et al., 2012), except that the GeneScan-LIZ500 size standard (Applied Biosystems, USA) was used in our assay.

### Bacterial virulence *in vivo*

Six-week-old BALB/c mice were purchased from the Wuhan Institute of Biological Products Co., Ltd. (Wuhan, China). All procedures and handling techniques were approved by the Laboratory Animal Monitoring Committee of Huazhong Agricultural University and were performed accordingly. We equally divided 30 female BALB/c mice into three groups: WT, Δ*cpxAR*, and CΔ*cpxAR*. Briefly, all strains were cultured in TSB at 37°C, harvested in logarithmic phase (OD_600_ = 0.6–0.8), and washed three times with PBS. For each group of test mice, bacteria at 1 × 10^7^ CFU mouse^−1^ were injected via the abdominal cavity, while an additional five control mice were injected with same volume of sterile PBS. The clinical signs, such as appetite, dyspnea, and lethargy, and mortality rates for each group were recorded twice daily.

To evaluate the colonization ability of Δ*cpxAR* in susceptible mouse tissue (lung), another 18 female BALB/c mice were divided into three equal groups (6 mice/group). Groups 1 and 3 were intraperitoneally administered 100 μL of PBS containing the WT strain (5 × 10^5^ CFU mouse^−1^) or the CΔ*cpxAR* strain (5 × 10^5^ CFU mouse^−1^), respectively, while group 2 received a dose of Δ*CpxAR* (6.5 × 10^5^ CFU mouse^−1^). After 72 h, the blood of each mouse was removed via cardiac perfusion, and the lung was aseptically removed. Each lung was homogenized (100 mg weight mL^−1^ of PBS) using a Tissuelyser (Jingxin, Shanghai, China), and 100 μL of the resulting homogenate were processed for determining the CFU counts.

### Bioinformatic and statistical analyses

The prediction of bacterial promoters was carried out using the tool in http://fruitfly.org/seq_tools/promoter.html. All data are shown as means ± standard deviation. The statistical analyses of the results were performed using a two-tailed Student's *t*-test or a one- or two-way analysis of variance, and comparisons with a *p* < 0.05 were considered as statistically significant. The data and analyses were graphed using GraphPad Prism.

## Results

### Bioinformatics analysis of the *cpxAR* genes and RT-PCR verification

**Figure 1:**
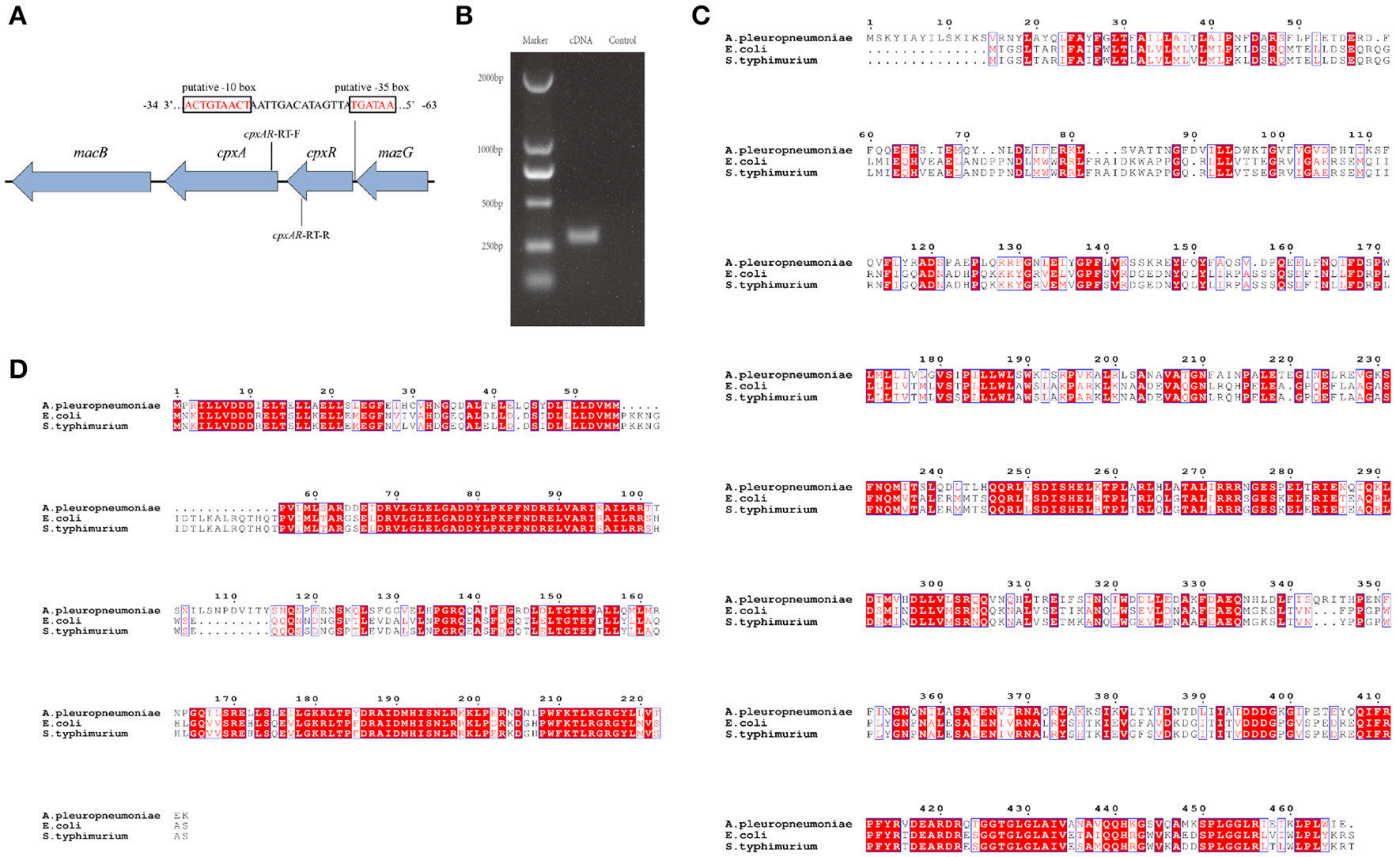
Schematic structure of the *cpxAR* genes, RT-PCR verification and Bioinformatics analysis. **(A)** Schematic structure of the *cpxA* and *cpxR* genes. There have 64 bp intergenic spacing between the *cpxA* and *cpxR* genes. The figure shows the genetic organization of genes up- and down-stream of *cpxAR*. There is a putative −10 box and a putative −35 box before the *cpxR* start codon. The promoter of the *cpxR* gene was predicted by http://fruitfly.org/seq_tools/promoter.html. The *E. coli* consensus for the −35 box is TTGACA and the −10 box is TATAAT (Shimada et al., 2014). The schematic is not to scale. **(B)** The primers *cpxAR*-RT-F/R were used to amplify the junction regions of *cpxA*–*cpxR* by RT-PCR. Multiple sequence alignments of CpxA **(C)** and CpxR **(D)** were performed using ClustalW2 (http://www.ebi.ac.uk/Tools/msa/clustalw2/).

To gain a thorough understanding of the role of CpxA/CpxR in *A. pleuropneumoniae*, we first analyzed the genetic organization of the *cpxAR* operon *in silico*. The results indicate that the *cpxA* and *cpxR* genes form an operon (Figures 1A,B). We also found that the CpxA and CpxR proteins of *A. pleuropneumoniae* each have a relatively high amino acid sequence identity with those of *E. coli* and *Salmonella enterica* serovar Typhimurium (Figures 1C,D). BlastP analysis revealed that the *A. pleuropneumoniae* CpxA has 41% amino acid sequence identity each with the CpxA from *E. coli* and *Salmonella enterica* serovar Typhimurium, while CpxR has 58 and 59% amino acid sequence identity, respectively, with the CpxR of these two species.

### Construction of the Δ*cpxAR* mutant and complemented strain CΔ*cpxAR*

An unmarked, in-frame mutant strain Δ*cpxAR* was designed based on the genome sequence of *A. pleuropneumoniae* strain S4074, along with a complemented strain CΔ*cpxAR* to confirm that there was no polarity effect in the Δ*cpxAR* using plasmid pJFF224-XN. The successful constructions of mutant strain Δ*cpxAR* and complemented strain CΔ*cpxAR* were confirmed by PCR using primers *cpxAR*-exterior F/R and *cpxAR*-interior F/R (Supplementary Figure 1) and by RT-PCR using primers cpxA-F/R and cpxR-F/R (Supplementary Figure 2). Furthermore, DNA sequencing results confirmed the sequence accuracy of these two strains (data not shown).

### CpxA/CpxR is required for biofilm formation

**Figure 2:**
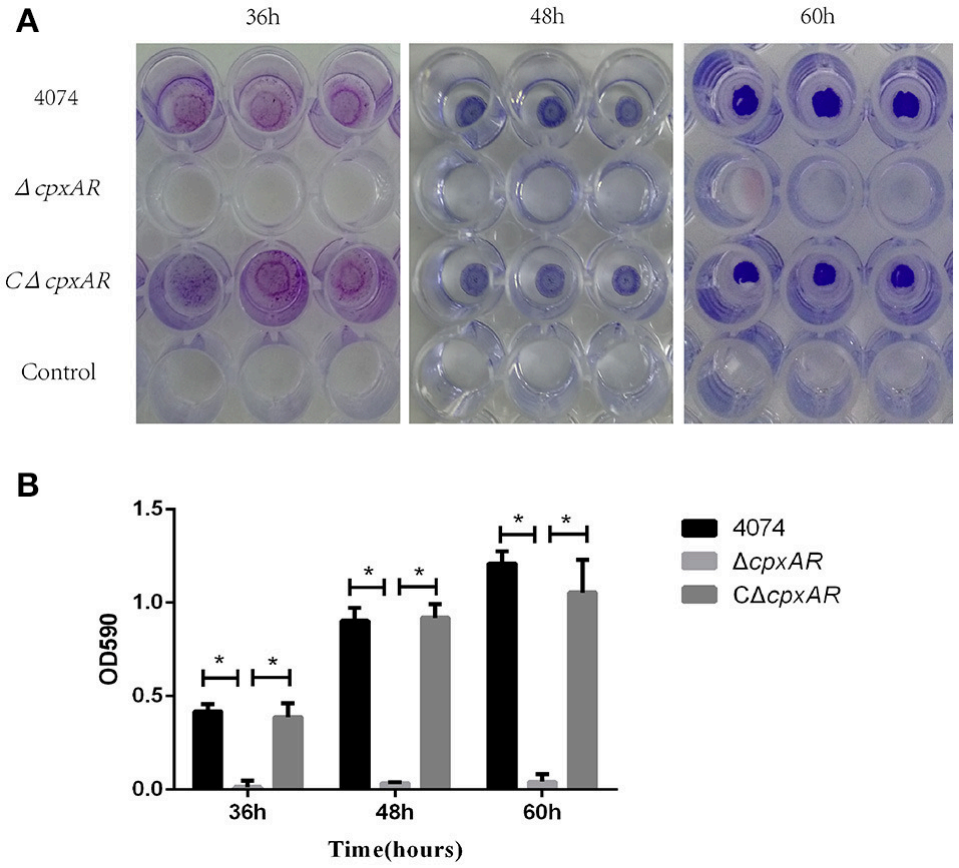
Polystyrene microtiter plate biofilm assay of *A. pleuropneumoniae* grown in TSB. **(A)** Biofilm formation of the S4074, Δ*cpxAR*, and CΔ*cpxAR* strains in the wells of 96-well polystyrene microtiter plates at 42°C. The plates were stained with crystal violet. **(B)** Quantitative determination of biofilm formation. The S4074, Δ*cpxAR*, and CΔ*cpxAR* strains were grown in TSB at 42°C. The optical density of the bacterial biofilm was monitored at OD_590_ after 36, 48, and 60 h of incubation. The data represents the mean ± S.D. of three independent experiments performed in duplicate. ^*^p < 0.05.

No biofilm growth was observed when the WT S4074 strain was grown in TSB at 37°C (Supplementary Figure 3), which confirms the results of previous studies (Kaplan and Mulks, 2005). Figure 2 illustrates the biofilm formation of WT, Δ*cpxAR*, and CΔ*cpxAR* at 42°C. At this temperature, the WT and CΔ*cpxAR* strains formed a pronounced biofilm that increased over time, whereas the mutant strain Δ*cpxAR* was unable to form any visible biofilm at 42°C at any time point (Figure 2A). A quantitative assay, which monitored the OD_590_, also revealed a significant difference between the Δ*cpxAR* mutant strain and the WT and CΔ*cpxAR* strains in their abilities to form a biofilm (Figure 2B). Additionally, we also compared biofilm formation by the WT and *cpxAR* mutant strain when the bacteria were grown for 24 h in BHIB at 37°C, which is the most frequently-used culturing condition. The results similarly show that the biofilm formed by the Δ*cpxAR* mutant was significantly smaller than those formed by the WT and CΔ*cpxAR* strains (Supplementary Figure 4).

### Growth analysis of the WT, Δ*cpxAR*, and CΔ*cpxAR* strains

**Figure 3:**
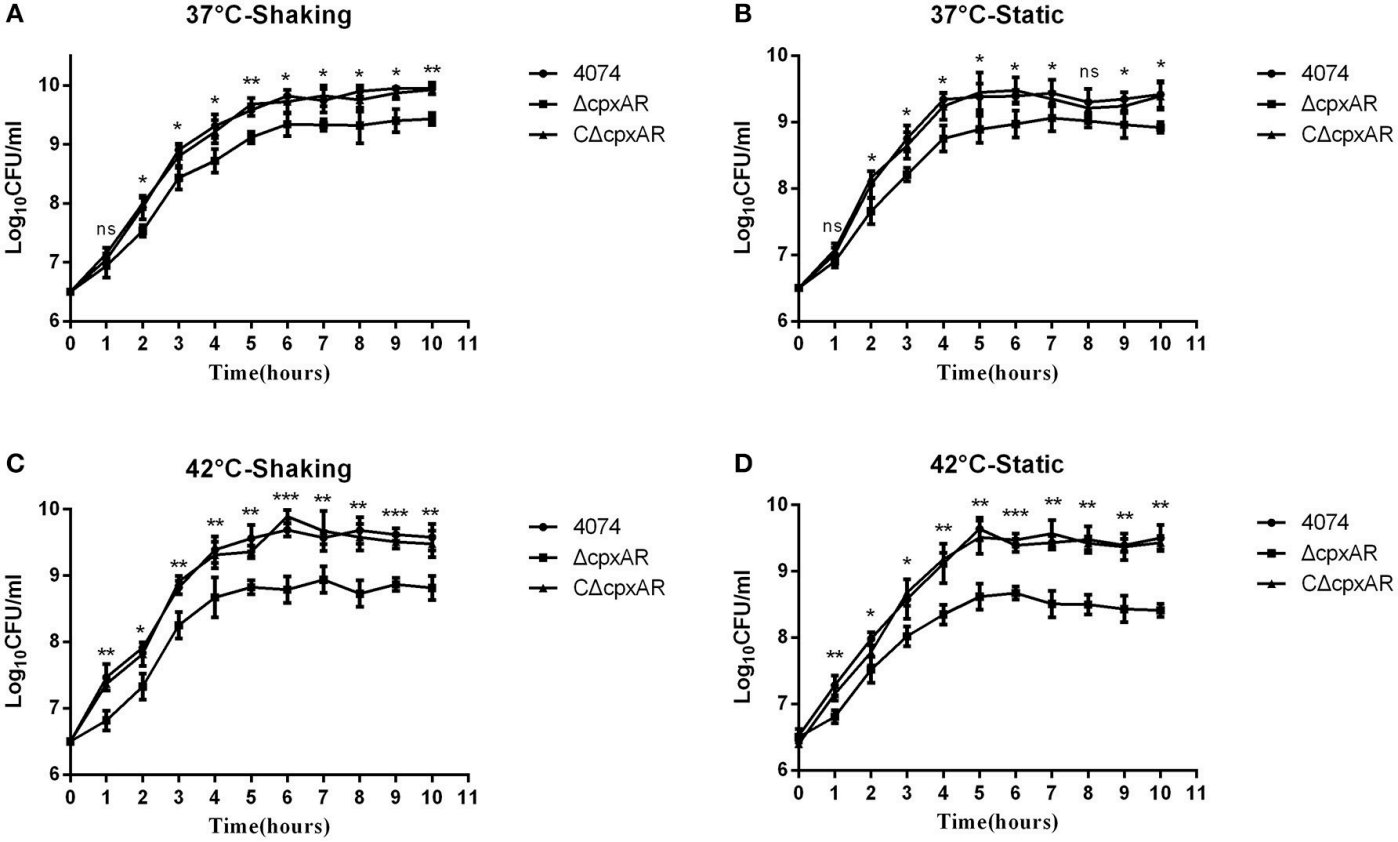
Growth curves of the WT, mutant, and complemented strains of *A. pleuropneumoniae*. Growth curves of the WT (S4074), mutant (Δ*cpxAR*), and complemented (CΔ*cpxAR*) strains of *A. pleuropneumoniae* at 37°C under shaking **(A)** or static **(B)** conditions or at 42°C under shaking **(C)** or static **(D)** conditions are shown. Bacterial growth was monitored by measurement of viable cell counts expressed as LogCFU/mL. The data represents the mean ± S.D. of three independent experiments performed in duplicate. ^***^p < 0.001, ^**^p < 0.01, ^*^p < 0.05.

The growth rates of the WT, Δ*cpxAR*, and CΔ*cpxAR* strains in TSB were analyzed at 37°C and 42°C under shaking and static conditions (Figure 3). A lower growth rate was observed with mutant strain Δ*cpxAR* compared with the WT and complemented mutant strains at both temperatures. The lack of a growth defect in CΔ*cpxAR* demonstrates that CpxA/CpxR slightly affects the growth rate and indicates that there likely were not any polar effects of the mutations or undetected mutations at other genetic loci.

### CpxA/CpxR regulates the mRNA levels of some important biofilm-related genes

**Figure 4:**
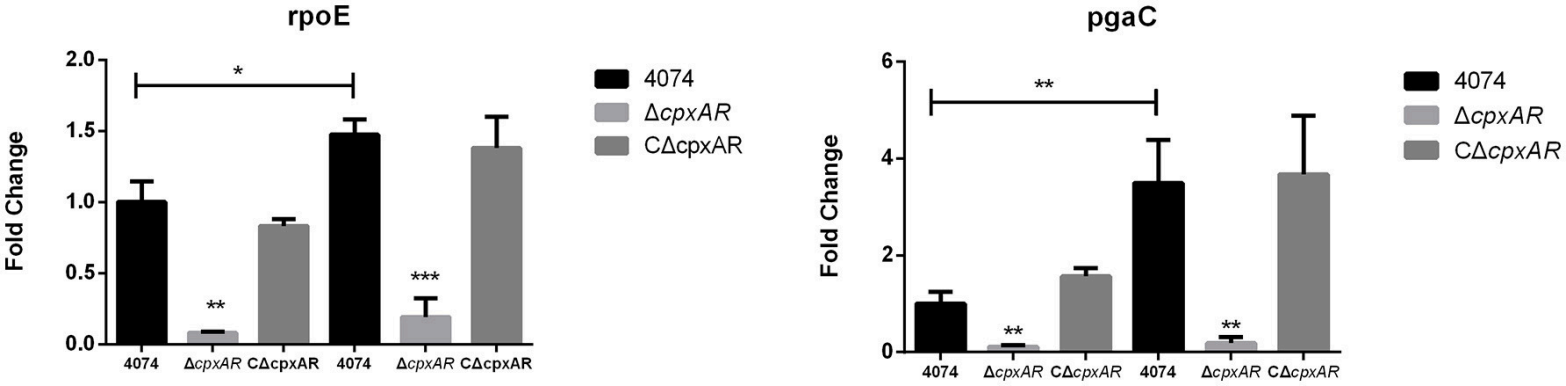
Effects of the *cpxA/cpxR* deletion on the transcription of biofilm-related genes. QRT-PCR analysis of the transcription of important biofilm-related genes (*pgaC* and *rpoE*) in the WT S4074, Δ*cpxAR*, and CΔ*cpxAR* strains at different temperatures. The mRNA levels of biofilm-related genes were normalized to those of the *16S RNA* gene for each strain. Data presented are the mean ± S.D. from three independent experiments performed in duplicate. ^***^p < 0.001, ^**^p < 0.01, ^*^p < 0.05.

Many biofilm-related genes have been identified in recent years, and selected important genes, such as *pgaC* (encoded within the *pgaABCD* operon; Kaplan et al., 2004) and *rpoE* (Bosse et al., 2010) were subsequently studied further. Our results indicate that the *pgaC* and *rpoE* genes were each expressed at lower levels in the mutant strain Δ*cpxAR* when grown at 42°C (Figure 4). Interestingly, there were also remarkably similar fold-changes in these genes at 37°C, even though Δ*cpxAR* was not able to form a biofilm at this temperature. Additionally, these results show that the expression levels of the *pgaC* and *rpoE* genes were each upregulated (*p* < 0.05) in the parental strain S4074 grown at 42°C compared with their expression levels when the bacteria were grown at 37°C.

### CpxR binds to the promoter region of the *rpoE* gene in *A. pleuropneumoniae*

**Figure 5:**
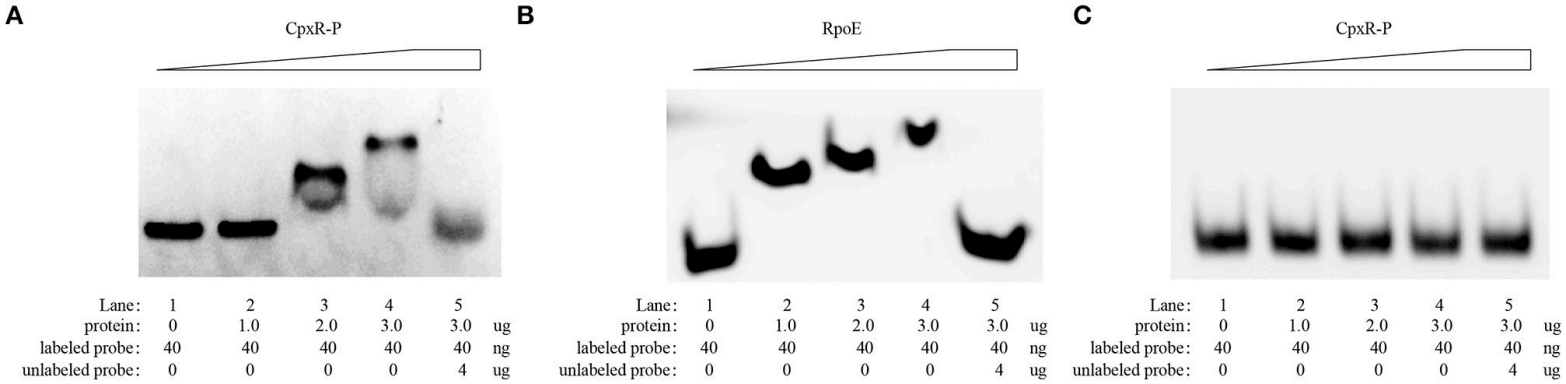
EMSA evaluation of the *in vitro* direct binding of CpxR-P and RpoE to the target promoter region of *rpoE* and *pga* respectively. An EMSA was performed to evaluate the *in vitro* direct binding of CpxR-P and RpoE to the target promoter region of *rpoE* **(A)** and *pga* **(B)**. Protein (0, 1.0, 2.0, and 3.0 μg) and FAM-labeled DNA fragments (40 ng) were added to the binding reaction. For the competition control, an excess amount of unlabeled competitor DNA (4 μg) was added to the reaction mixture. **(C)** Binding of CpxR-P to the target promoter region of *pga* was negative control.

**Figure 6:**
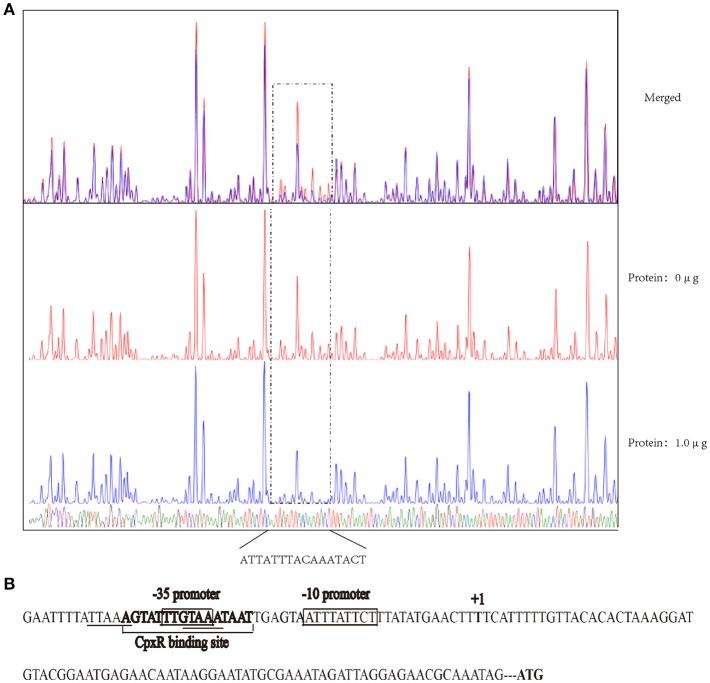
DNase I footprinting assay assessing the *in vitro* direct binding of CpxR-P to the promoter region of *rpoE*. **(A)** A DNase I footprinting assay of the *rpoE* promoter DNA fragment was performed in the absence or presence of CpxR-P. The FAM-labeled 273-bp DNA fragments (400 ng) that had been pre-incubated in the absence or presence of 1.0 μg of CpxR-P were subjected to DNase I digestion and a subsequent fragment length analysis. The fluorescent signals for the labeled DNA fragments are shown plotted against the sequences of the fragments. **(B)** Summary of the results from the DNase I footprinting assay described above. The sequence of the predicted motif is shown with the putative core regions underlined and the sequence determined by the DNase I footprinting assay in bold. The promoter region of *rpoE* gene is shown enclosed in a box.

The recognition site of phosphorylated CpxR (CpxR-P) has a conserved sequence GTAAA-(N)_4−8_-GTAAA (Yamamoto and Ishihama, 2006; Srinivasan et al., 2012; Bernal-Cabas et al., 2015). SDS-PAGE and Western blot results show that the His-CpxR and His-RpoE were successfully expressed in supernatant (Supplementary Figure 5). EMSAs revealed that CpxR-P and RpoE clearly bound to the promoter region of the *rpoE* gene and the *pga* operon respectively in a dose-dependent manner, and these binding were abolished by adding excess unlabeled competitor DNA (Figure 5). Furthermore, a DNase I footprinting analysis also revealed that the promoter region of *rpoE* had a CpxR-P-binding site (5′-AGTATTTGTAAATAAT-3′), which was 25 bp upstream of the putative transcription start site (Figure 6A and Supplementary Figure 7). The CpxR-P-binding site partially coincided with the conserved sequence GTAAA-(N)_4−8_-GTAAA, and overlapped the promoter −35 region (Figure 6B). These data show that CpxR-P binds directly to the promoter region of *rpoE*. Taken together, these data support that under this biofilm formation condition (42°C, TSB, static), CpxR-P can regulate the expression of the *pga* operon through *rpoE*.

### Virulence of the *cpxAR* mutant in mice

**Figure 7:**
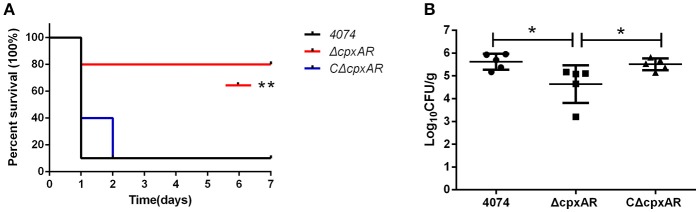
Survival curves and lung bacteria loads of mice infected with *A. pleuropneumoniae*. **(A)** Survival curves for Balb/c mice infected with the WT, Δ*cpxAR*, and *C*Δ*cpxAR* strains. Six-week-old Balb/c mice were inoculated intraperitoneally with 1 × 10^7^ CFU of bacteria, and their survival was monitored over a 7 day period. Data were analyzed using the log rank test. **(B)** The *A. pleuropneumoniae* load in the lungs of infected mice. The horizontal lines denote the median number of bacteria in each group of 6 mice. The data is representative of one of three independent experiments. ^**^p < 0.01, ^*^p < 0.05.

The role of CpxA/CpxR in the virulence of *A. pleuropneumoniae* was evaluated *in vivo* in BALB/c mice, which serve as an appropriate model for *A. pleuropneumoniae* infection (Chiang et al., 2009; Seo et al., 2013; Yuan et al., 2014; Wang et al., 2015; Xie et al., 2016). As shown in Figure 7A, 90% of the mice in the WT and CΔ*cpxAR* groups died within 7 days, but only two mice in the Δ*cpxAR* group died within this time frame. Overall, the survival rate of the Δ*cpxAR* group was significantly higher than those of the WT and CΔ*cpxAR* groups (*p* < 0.05).

As shown in Figure 7B, the amount of colonization by the Δ*cpxAR* strain in the lungs was significantly lower compared with those by the WT and CΔ*cpxAR* strains (*p* < 0.001). This result reveals that the CpxA/CpxR TCS is necessary for *A. pleuropneumoniae* colonization *in vivo*. Taken together, these results indicate that the CpxA/CpxR TCS contributes to the virulence of *A. pleuropneumoniae*.

## Discussion

Although many studies regarding biofilm formation in *A. pleuropneumoniae* have cited *cpxAR* and consider *cpxAR* to be a biofilm-related factor, no reports have directly validated the function of *cpxAR* in *A. pleuropneumoniae* (Tremblay et al., 2013; Hathroubi et al., 2015a,b). A mutation of *cpxAR* in *A. pleuropneumoniae* resulted in a lower growth rate than observed for the corresponding WT strain.

Previous work showed that *A. pleuropneumoniae* strain S4074 can produce a pronounced biofilm in BHI broth that had lower than normal levels of zinc, but this condition is inconvenient for use in research (Labrie et al., 2010). In this study, our microtiter plate biofilm assays revealed that although S4074 cannot produce a detectable biofilm at 37°C, it does produce a pronounced biofilm at 42°C. Furthermore, the qRT-PCR results revealed that the expression levels of three biofilm-related genes (*rpoE* and *pgaC*) in S4074 were significantly upregulated at 42°C compared with their expression levels at 37°C. Thus, our qRT-PCR results were consistent with the results of the biofilm assays. In addition, the expression of *cpxR* was upregulated in S4074 at 42°C compared with at 37°C (Supplementary Figure 6). Together, these results indicate that high temperature can induce a pronounced biofilm through upregulating the *cpxR* gene in S4074.

CpxR was previously shown to bind to the promoter region of the regulator σ^E^ in *E. coli* (De Wulf et al., 2002). In this study, qRT-PCR revealed that CpxA/CpxR positively influences transcription of the *rpoE* gene in *A. pleuropneumoniae*. Additionally, the sequence GTAAA-(N)_4−8_-GTAAA is the CpxR-binding site in many other bacteria (Feldheim et al., 2016; Tian et al., 2016). Here, an *in silico* assessment identified a putative CpxR-binding sequence in the vicinity of the promoter region of sigma factor *rpoE*. We also demonstrated via EMSA that CpxR can directly bind to the promotor region of the *rpoE* gene. Furthermore, our DNase I footprinting analysis found that the CpxR-binding site was partially consistent with the putative sequence and was 25 bp away from the putative transcription start site. The CpxR-binding site is generally within 100 bp in the 5′ direction from the transcriptional start site, which is essential for CpxR-dependent activation of the target promoters in *E. coli* (De Wulf and Lin, 2000; Raffa and Raivio, 2002). Previous studies demonstrated that CpxR primarily functions as a class I factor binding upstream of the promoter −35 region in *E. coli* (Yamamoto and Ishihama, 2006). In this study, we observed that CpxR activates *rpoE*, as the CpxR binding site overlaps the −35 box sequence in *A. pleuropneumoniae*.

Notably, in TSB, the *cpxAR* mutant showed a remarkable fold-change in the expression of the *pgaC* gene, which was lower than that in the WT strain. Previous studies found that RpoE positively regulates the transcription of the *pga* operon, and the *rpoE* promoter sequence (GAACTT-n16-TCAAA) is 468 bp upstream from the *pgaA* translational start in *A. pleuropneumoniae* (Bosse et al., 2010). PgaC synthesizes the major biofilm matrix polysaccharide PGA, which is a component of *A. pleuropneumoniae* biofilm colonies and may prevent the access of antimicrobial agents to the bacterial cells within the biofilm (Costerton et al., 1999; Kaplan et al., 2004). In this study, we also demonstrated via EMSA that RpoE can directly bind to the promotor region of the *pga* operon. In light of these previous findings and those in the present study, we demonstrated that the CpxA/CpxR TCS in *A. pleuropneumoniae* is able to regulate the expression of the *pga* operon through *rpoE* to facilitate biofilm formation.

Our experimental results suggest that *cpxAR* could be a virulence-associated gene and, therefore, may be a potential therapeutic target (Wassenaar and Gaastra, 2001). Many major virulence factors reside in the cell envelope, so it is reasonable that the Cpx response is involved in the development of host infection caused by various bacterial pathogens (Vogt and Raivio, 2012). Additionally, RpoE plays an important role in the virulence of *Salmonella* Typhimurium, *Burkholderia pseudomallei, Vibrio cholera*, and *Vibrio harveyi* (Humphreys et al., 1999; Kovacikova and Skorupski, 2002; Thongboonkerd et al., 2007; Rattanama et al., 2012). Furthermore, it has been observed that the *pgaBC* genes were upregulated in *A. pleuropneumoniae* following contact with St. Jude porcine lung cells (Auger et al., 2009). In this study, the colonization assay and survival results demonstrate that CpxA/CpxR contributes to the virulence of *A. pleuropneumoniae*. Taken together, these results indicate that the regulation of biofilm formation by CpxA/CpxR may be involved in the pathogenesis of *A. pleuropneumoniae*.

Biofilm formation is reportedly involved in escaping from the host immune system, thereby playing an important role in the pathogenicity of *A. pleuropneumoniae in vivo* (Bosse et al., 2010; Hathroubi et al., 2017). The predominant sign of acute pleuropneumonia is high fever that body temperature can elevated up to 41.8°C (Menzel et al., 2014; Sassu et al., 2017). Here we demonstrate that the fever temperature contribute to biofilm formation in *A. pleuropneumoniae*. Therefore, the mechanism may promote the understanding of the pathogenesis of *A. pleuropneumoniae*-induced infection.

## Author contributions

WB, HL, FL conceived and designed the experiments. HL, KY, WP, HZ, HuiC, and TL performed the experiments. FL, HL, PC analyzed the data. FY, HuaC contributed reagents, materials and analysis tools.

### Conflict of interest statement

The authors declare that the research was conducted in the absence of any commercial or financial relationships that could be construed as a potential conflict of interest.

## Supplementary material

Supplementary Figure 1 Identification of the *A. pleuropneumoniae* mutant strain and the complemented strain by PCR. Identification of the Δ*cpxAR* mutant strain and the CΔ*cpxAR* complemented strain by PCR, using PCR primer pairs *cpxAR*-exterior F/R (2,935 bp for WT, 573 bp for Δ*cpxAR* and CΔ*cpxAR*) and *cpxAR*-interior F/R (506 bp for WT and CΔ*cpxAR*, no fragment for Δ*cpxAR*).

Supplementary Figure 2 RT-PCR analysis of *cpxAR* transcripts. Total RNA was extracted from the WT, Δ*cpxAR*, and CΔ*cpxAR* strains. cDNA generated from these RNA samples was subjected to RT-PCR analysis with primer pairs cpxA-F/R and cpxR-F/R. The products were analyzed by electrophoresis. The DL 2000 DNA Marker is shown on the left (lane M).

Supplementary Figure 3 Polystyrene microtiter plate biofilm assay of *A. pleuropneumoniae* grown in TSB at 37°C. The S4074, Δ*cpxAR*, and CΔ*cpxAR* strains were grown in TSB at 37°C. The resulting bacterial biofilms were photographed **(A)** and measured **(B)** at OD_590_ after 36, 48, and 60 h of incubation. Each data point represents the mean ± S.D. from three independent experiments performed in duplicate.

Supplementary Figure 4 Polystyrene microtiter plate biofilm assay of *A. pleuropneumoniae* grown in BHI at 37°C for 24 h. The S4074, Δ*cpxAR*, and CΔ*cpxAR* strains were grown in BHI at 37°C. The optical density of the bacterial biofilm was monitored at OD_590_ after 24 h of incubation. Each data point represents the mean ± S.D. from three independent experiments performed in duplicate. ^*^*p* < 0.05.

Supplementary Figure 5 Identification of purified His-CpxR protein and His-RpoE protein. Sodium dodecyl sulfate polyacrylamide gel electrophoresis behavior **(A,C)** and Western blot analyses **(B,D)** of the purified His-CpxR protein and His-RpoE protein respectively.

Supplementary Figure 6 Effects of temperature on the transcription of *cpxAR* genes. A qRT-PCR analysis of *cpxAR* gene transcriptions in the WT S4074 strain at different temperatures is shown. The mRNA levels of *cpxAR* genes were normalized to those of the *16S RNA* gene. Data presented are the mean ± S.D. from three independent experiments performed in duplicate. ^*^*p* < 0.05.

Supplementary Figure 7 DNase I footprinting assay. The FAM-labeled 273-bp DNA fragments (400 ng) that had been pre-incubated with 1.0 μg **(A)** or 2.0 μg **(B)** of CpxR-P were subjected to DNase I digestion and a subsequent fragment length analysis.
