# Supplementary figures and images for: The CpxA/CpxR Two-Component System Affects Biofilm Formation and Virulence in Actinobacillus pleuropneumoniae
Source: Front Cell Infect Microbiol. 2018 Mar 20;8:72. doi: 10.3389/fcimb.2018.00072 (PMC5890194; doi:10.3389/fcimb.2018.00072)

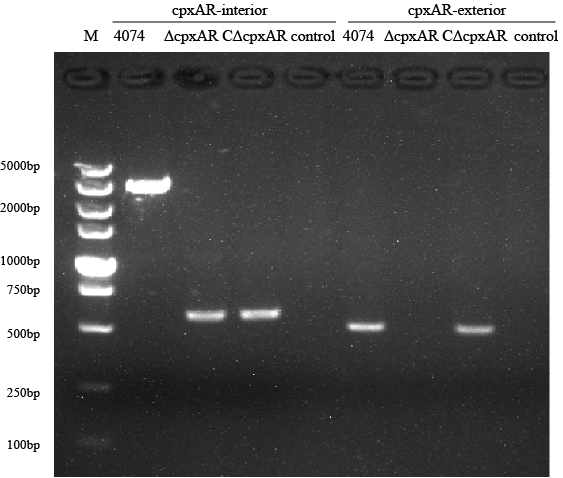

Supplement: Supplementary Figure 1 — Identification of the A. pleuropneumoniae mutant strain and the complemented strain by PCR. Identification of the ΔcpxAR mutant strain and the CΔcpxAR complemented strain by PCR, using PCR primer pairs cpxAR-exterior F/R (2,935 bp for WT, 573 bp for ΔcpxAR and CΔcpxAR) and cpxAR-interior F/R (506 bp for WT and CΔcpxAR, no fragment for ΔcpxAR). [file Image1.TIF]

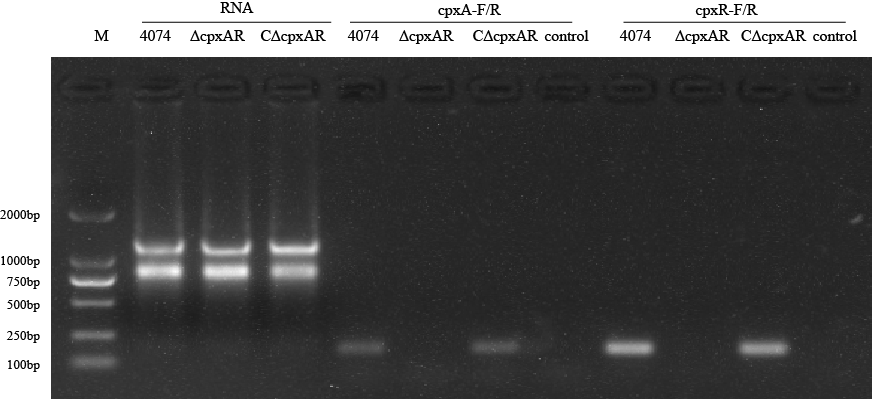

Supplement: Supplementary Figure 2 — RT-PCR analysis of cpxAR transcripts. Total RNA was extracted from the WT, ΔcpxAR, and CΔcpxAR strains. cDNA generated from these RNA samples was subjected to RT-PCR analysis with primer pairs cpxA-F/R and cpxR-F/R. The products were analyzed by electrophoresis. The DL 2000 DNA Marker is shown on the left (lane M). [file Image2.TIF]

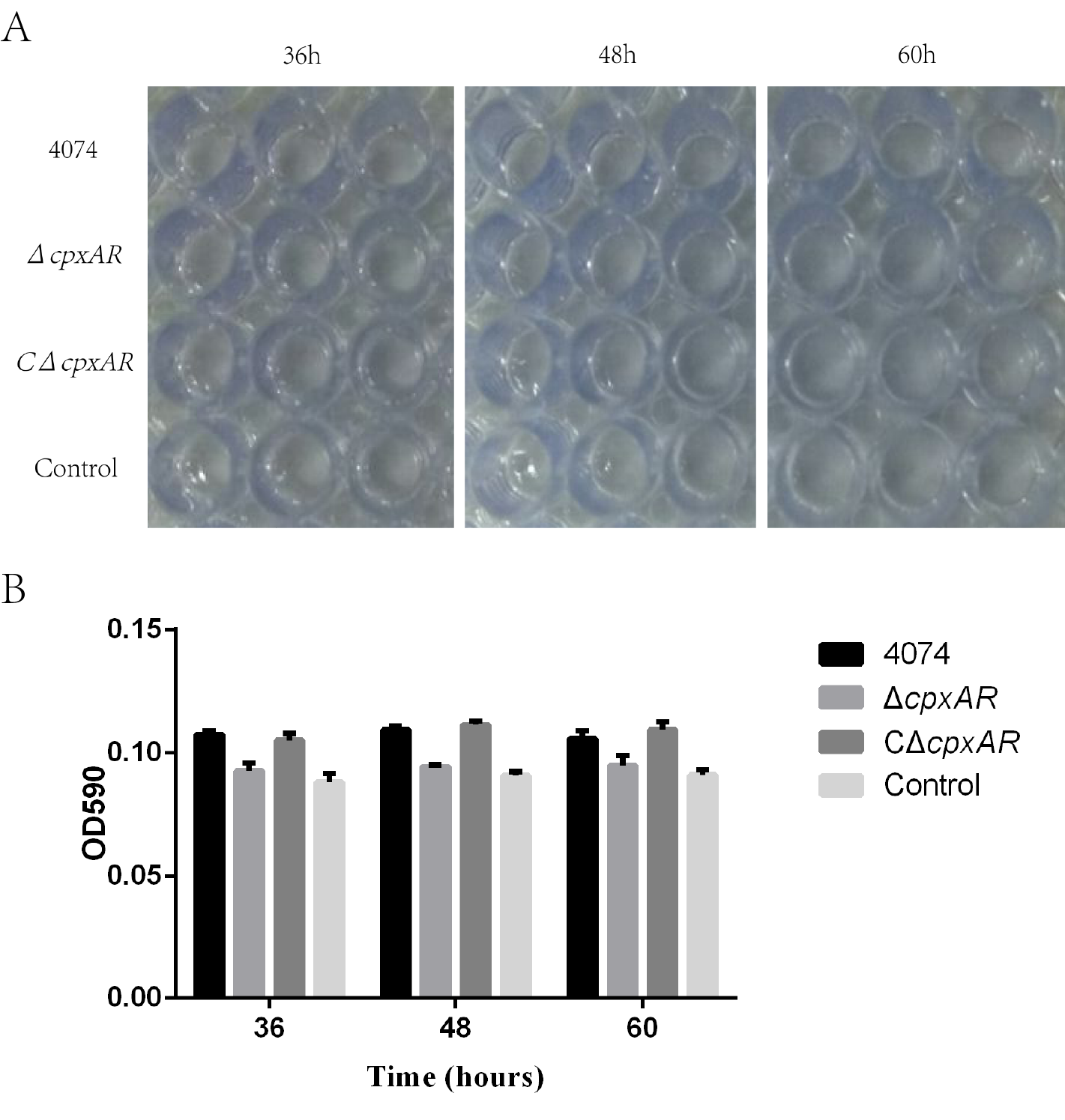

Supplement: Supplementary Figure 3 — Polystyrene microtiter plate biofilm assay of A. pleuropneumoniae grown in TSB at 37°C. The S4074, ΔcpxAR, and CΔcpxAR strains were grown in TSB at 37°C. The resulting bacterial biofilms were photographed (A) and measured (B) at OD590 after 36, 48, and 60 h of incubation. Each data point represents the mean ± S.D. from three independent experiments performed in duplicate. [file Image3.TIF]

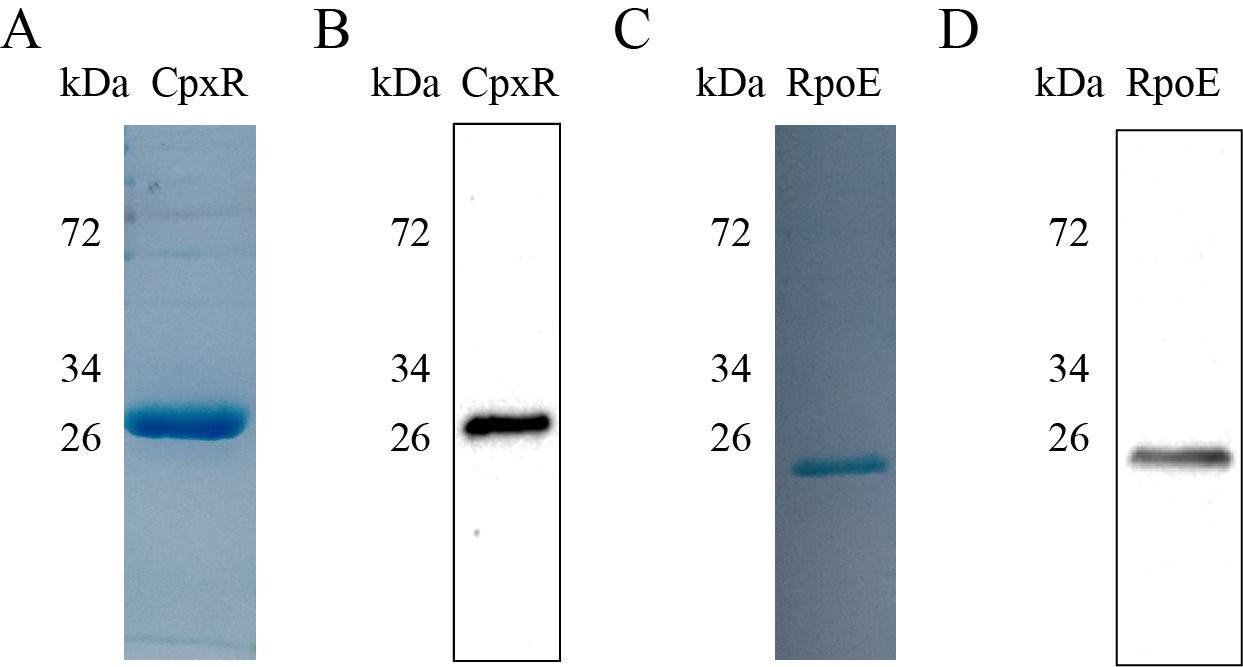

Supplement: Supplementary Figure 5 — Identification of purified His-CpxR protein and His-RpoE protein. Sodium dodecyl sulfate polyacrylamide gel electrophoresis behavior (A,C) and Western blot analyses (B,D) of the purified His-CpxR protein and His-RpoE protein respectively. [file Image5.TIF]

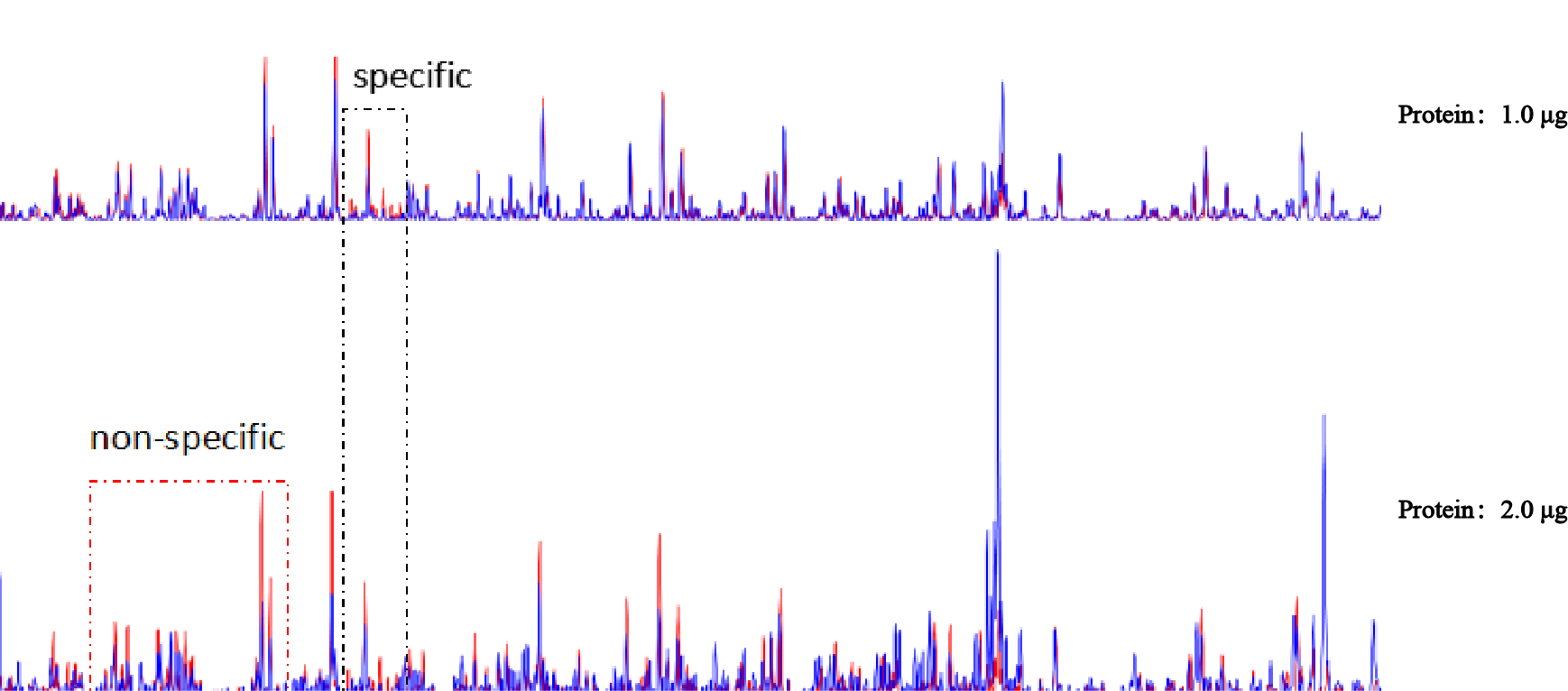

Supplement: Supplementary Figure 7 — DNase I footprinting assay. The FAM-labeled 273-bp DNA fragments (400 ng) that had been pre-incubated with 1.0 μg (A) or 2.0 μg (B) of CpxR-P were subjected to DNase I digestion and a subsequent fragment length analysis. [file Image7.tif]
